# Epidemiology and treatment in patients with HIV in a secondary-level hospital in Mexico

**DOI:** 10.17843/rpmesp.2025.424.14693

**Published:** 2025-12-10

**Authors:** María Fernanda Aguilar-Etchegaray, Hector Martinez-Troncoso, Lilia Susana Gallardo-Vidal, Brenda Michelle Pérez-García, Jacqueline Jiménez-Avila

**Affiliations:** 1 Family Medicine Unit No. 13, Mexican Social Security Institute, Querétaro, Mexico. Mexican Social Security Institute Family Medicine Unit No. 13 Mexican Social Security Institute Querétaro Mexico; 2 Regional General Hospital No. 1, Mexican Social Security Institute, Querétaro, Mexico. Mexican Social Security Institute Regional General Hospital No. 1 Mexican Social Security Institute Querétaro Mexico

**Keywords:** Epidemiology, HIV, Antiretroviral Therapy, Highly Active

## Abstract

To describe the sociodemographic, clinical, and therapeutic characteristics of patients with Human Immunodeficiency Virus (HIV) at a second-level hospital in Mexico, a descriptive study was conducted based on the review and analysis of 871 clinical records from 2017 to 2023. The results show a predominance of the male sex (91.5%) with ages between 30 and 39 years (45.5%), and a homosexual sexual orientation (68.0%). About 25.4% had an initial CD4 count of <200 cells, 99% received antiretroviral therapy (ART), and subsequently 54.5% had a CD4 count of >500 cells, 88.1% presented with an undetectable viral load, and 1.6% experienced treatment failure. In conclusion, the young, single, homosexual male population is vulnerable to HIV infection. These findings highlight the need to strengthen prevention programs, timely diagnosis, and early treatment, to improve clinical outcomes and reduce HIV transmission.

## INTRODUCTION

Despite the significant epidemiological changes in recent years regarding Human Immunodeficiency Virus (HIV) infection, it remains a public health problem globally ^1^ and in Mexico ^2^^-^^4^. Faced with this situation, the World Health Organization’s UNAIDS (Joint United Nations Programme on HIV/AIDS) instituted specific improvement objectives for patients with HIV for the year 2020, called the “90/90/90 goals,” to ensure that 90% of people have a serological diagnosis, receive antiretroviral treatment (ART), and achieve an undetectable viral load ^5^^,^^6^.

Despite the progress made with ART, the widespread implementation of single-tablet regimens and long-acting injectable management ^3^ has been hampered. Although these drugs are considered more effective, better tolerated, and promote greater adherence, their introduction has not yet made it possible to achieve the proposed final goal.

This situation is reflected in the 2022 data, where only: 86% of people knew their serological status; 76% had access to ART; 71% had an undetectable viral load ^5^. In Mexico, one of the biggest challenges in facing HIV is timely diagnosis and treatment; unfortunately, coverage is only between 80% and 85%. Therefore, one of the major challenges in addressing HIV in Mexico is timely detection and ensuring that all people living with it are on ART ^2^.

According to UNAIDS, in Mexico, the populations with the highest incidence are the most vulnerable, such as men who have sex with men (MSM), transgender people (TG), sex workers, those who use intravenous drugs, and people deprived of their liberty ^6^^-^^8^, similar to populations in other countries ^4^^,^^8^. In most cases, diagnosis and treatment are not provided in a timely manner, which leads to advanced stages of immunosuppression, a poorer response to ART, and an increased risk of mortality ^9^.

Given these challenges, the Mexican Social Security Institute (IMSS) treated 82,716 patients infected with HIV in 2021; of these, 97.4% received ART and had a viral load of less than 1000 copies ^2^. In 2022, guidelines for health personnel were established to standardize the care of patients with HIV, optimize processes, and ensure quality of care through Comprehensive Care protocols ^2^. Nevertheless, it is necessary to develop more research that delves into the epidemiological aspects, timely diagnosis, and treatment of these patients. In this context, the objective of the present study was to describe the epidemiological and therapeutic characteristics of patients with HIV in a secondary-level hospital in Mexico.

**KEY MESSAGES**

Motivation for the study. According to the UNAIDS objectives, 90% of people living with HIV should know their serological status, receive antiretroviral treatment (ART), and achieve an undetectable viral load. Few current studies in Mexico evaluate these indicators.

Main findings. The young male population is more vulnerable to HIV infection. Likewise, heterosexual women constitute a risk group in relation to the mechanisms of virus transmission in Mexico. 99% of participants received ART and 88% achieved an undetectable viral load.

Implications. These findings expand current knowledge about HIV behavior in the population of Querétaro, Mexico, and underscore the importance of prevention and timely diagnosis.

## THE STUDY

A descriptive, cross-sectional study was conducted using the medical records of patients with HIV who received specialized care at the HIV and AIDS Prevention and Control Clinic at Regional General Hospital No. 1 in the State of Querétaro, Mexico, during the period from 2017 to 2023. Patients aged 18 years or older with a confirmed HIV diagnosis were included; 5 records were excluded.

The variables analyzed were age, marital status, education level, sex, and sexual orientation, comorbidities, and history of diseases; CD4 cell count/uL (low <200; medium 201-499; high >500 or without laboratories), viral load (undetectable <40 copies/mL; detected; failure; without treatment; without laboratories), both based on the most recent result registered in the notes and records system, time from HIV diagnosis (<2 years; 3-5 years; 6-11 years or >11 years), and initiation of treatment (<1 month from diagnosis; 1 to 12 months).

The results were analyzed using frequencies and percentages via the IBM SPSS Statistics version 2019 program. The study protocol was approved by the Ethics and Research Committee of the IMSS at Regional General Hospital No. 1 (HGR N°1) with folio: R-2024-2201-116. Patients’ personal data were protected through the use of a numerical identifier. The authors declare that no identifiable patient data appear in this article.

## FINDINGS

Of 871 HIV cases identified at HGR N°1 of IMSS Querétaro, Mexico, from 2017 to 2023. Analysis of characteristics by sex showed male predominance 797 (91.5%), single marital status (74.8%), age 30-39 years (45.5%), bachelor’s degree education (39.8%), employed occupation (61.5), homosexual sexual orientation (68.0%). In females, the characteristics were married marital status (28.4%), age 40-49 years (37.8%), secondary education (31.1%), homemaker occupation (40.5%), heterosexual sexual orientation (91.9%).

Regarding comorbidities, infectious diseases 503 (57.75%), cutaneous diseases 260 (29.85%), and psychiatric diseases 181 (20.78%) preponderated. A predominance of oncological diseases (27.2%), diabetes (22.97%), and endocrinopathy (12.16%) was observed in females compared to males (Table 1).

**Table 1:** Sociodemographic characteristics and comorbidities found in patients with HIV by sex.

Characteristics (n = 871)		Total		Men		Women	
		n	%	n	%	n	%
Marital status							
	Not specified	68	7.8	61	7.7	7	9.5
	Single	616	70.7	600	75.1	16	21.5
	Married	75	8.6	54	6.8	21	28.4
	Consensual union	86	9.9	69	8.7	17	23.0
	Divorced	7	0.8	6	0.8	1	1.4
	Widowed	19	2.2	7	0.9	12	16.2
Age group							
	20-29	109	12.5	105	13.2	4	5.4
	30-39	378	43.3	363	45.5	15	20.3
	40-49	191	21.9	163	20.5	28	37.8
	50-59	123	14.1	107	13.4	16	21.6
	>60	70	8.0	59	7.4	11	14.9
Education level							
	Illiterate	82	9.4	68	8.5	14	18.9
	Elementary school	35	4.0	23	2.9	12	16.2
	High school	118	13.6	95	11.9	23	31.1
	High school diploma	286	32.8	266	33.4	20	27.0
	Technical degree	29	3.3	28	3.5	1	1.1
	Bachelor’s degree	321	36.9	317	39.8	4	5.4
Occupation							
	Homemaker	84	9.6	54	6.8	30	40.5
	Worker	65	7.5	57	7.2	8	10.8
	Employee	518	59.5	490	61.5	28	37.8
	Professional	156	17.9	154	19.3	2	2.7
	Merchant	29	3.3	24	3.0	5	6.8
	Student	19	2.2	18	2.3	1	1.4
Sexual orientation							
	Heterosexual	142	16.3	74	9.3	68	91.9
	Homosexual	543	62.3	542	68.0	1	1.4
	Bisexual	59	6.7	59	7.4	0	0.0
	Not specified	127	14.5	122	15.3	5	6.8
Comorbidties / diseases							
	Hypertension	87	9.9	76	9.54	11	11.8
	Diabetes	63	7.2	46	5.77	17	22.9
	Nephropathy	61	7.0	57	7.15	4	5.4
	Endocrinopathy	56	6.4	47	5.9	9	12.1
	Psychiatric	181	20.7	163	2.5	18	24.3
	Oncological	79	9.0	59	7.4	20	27.0
	Cutaneous	260	29.8	236	29.6	24	32.4
	History of tuberculosis	42	4.8	40	5.0	2	2.7
	Infectious diseases	503	57.7	462	57.9	41	55.4

The most frequent type of cancer in females was cervical cancer (CC) at 78.9% and in males it was Kaposi’s sarcoma at 35.4%.

High CD4 count predominated in 54% of patients, followed by medium count in 35.4%, and undetectable viral load in 88.1%. 92.8% were managed through combined ART of nucleoside reverse transcriptase inhibitors (NRTIs) + non-nucleoside reverse transcriptase inhibitors (NNRTIs) + Integrase Inhibitor (INI) in a single tablet, followed by the NRTI + NNRTI combination in 1.6% of patients (Table 2).

**Table 2:** CD4 count, viral load, and antiretroviral treatment received by HIV patients, by sex.

		Total		Men		Women	
		n	%	n	%	n	%
Initial CD4 Count							
	Low (<200)	221	25.4	204	25.6	17	23.0
	Medium (201-499	219	25.1	199	25.0	20	27.0
	High (>500)	105	12.1	99	12.4	6	8.1
	Without laboratory results	326	37.4	295	37.0	31	41.9
	Last CD4Count						
	Low (<200)	65	7.5	59	7.4	6	8.1
	Medium (201-499)	308	35.4	281	35.2	27	36.5
	High (>500)	475	54.5	438	55.0	37	50.0
	Without laboratory results	23	2.6	19	2.4	4	5.4
Viral Load							
	Detected	66	7.6	60	7.5	6	8.1
	Undetectable	767	88.1	703	88.2	64	86.5
	Failure	14	1.6	13	1.6	1	1.4
	Without treatment	2	0.2	2	0.3	0	0.0
	Without laboratory results	22	2.5	19	2.4	3	4.1
Single Tablet							
	BET/ABC (2 ITRAN+INI)	808	92.8	742	93.1	66	89.2
Multiple Tablets							
	ITRAN+ITRNN+INI	2	0.2	2	0.3	0	0.0
	RITO+INI+MVC	2	0.2	2	0.3	0	0.0
	ITRAN+IP	5	0.6	4	0.5	1	1.4
	ITRNN+IP+INI	1	0.1	0	0.0	1	1.4
	RITO+INI	1	0.1	1	0.1	0	0.0
	IP+RITO+INI+MVC	2	0.2	2	0.3	0	0.0
	ITRAN+IP+RITO	10	1.1	10	1.3	0	0.0
	ITRAN+ITRNN	14	1.6	13	1.6	1	1.4
	2 ITRAN + IP	2	0.2	2	0.3	0	0.0
	2 ITRAN+IP+RITO+INI	1	0.1	1	0.1	0	0.0
	ITRAN+ITRNN+IP	8	0.9	8	1	0	0.0
	ITRAN+IP+INI	4	0.5	2	0.3	2	2.7
	2 ITRAN+ITRNN	5	0.6	4	0.5	1	1.4
Not taking medication		6	0.7	4	0.5	2	2.7

ITRAN: Nucleoside Reverse Transcriptase Inhibitor. ITRNN: Non-nucleoside Reverse Transcriptase Inhibitor. INI: Integrase Inhibitor. IP: Protease Inhibitor. RITO: Ritonavir. MVC: Maraviroc.

**Figure 1:**
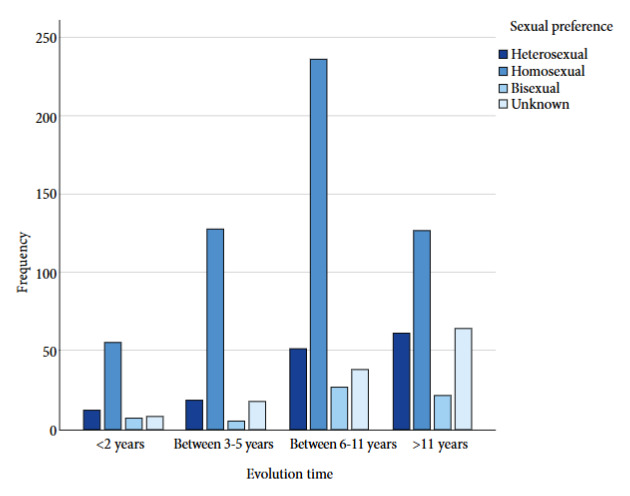
Time since diagnosis in patients diagnosed with HIV by sexual preference.

**Figure 2:**
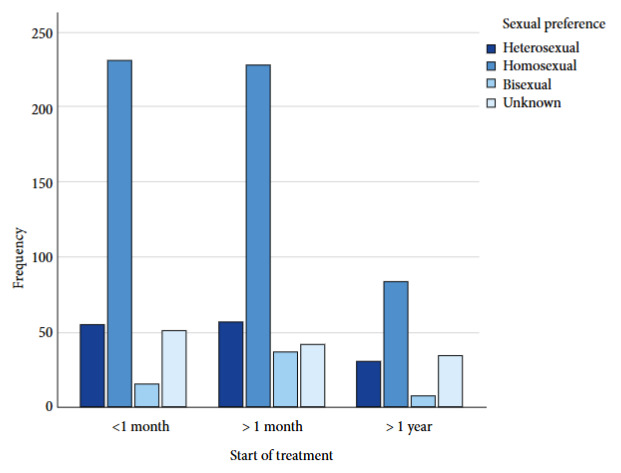
Initiation of antiretroviral treatment in HIV patients by sexual preference

An analysis by sexual preference and time since HIV diagnosis was performed, and it was observed that patients with homosexual preference 235 (43.3%), and 26 (44.1%) with bisexual preference, had a time since diagnosis of 6 to 11 years. In the case of those with heterosexual preference, 61 (43%) had a time since diagnosis of more than 11 years (Figure 1). Likewise, the relationship between ART initiation and sexual preference was analyzed, and it was observed that in the case of homosexual preference 232 (42.7%), and 54 (38%) patients with heterosexual preference initiated treatment before one month of diagnosis (Figure 2).

## DISCUSSION

The findings of the present study show that the male sex with homosexual orientation continues to be the most frequent group among patients with HIV. When analyzing the data by sex, it is observed that, among women, those who are married, homemakers, and older than men, but with a lower educational level, predominate.

Currently, there is little literature on the factors associated with HIV transmission in heterosexual women, which limits the understanding of the characteristics of this population ^10^^-^^12^. This group is considered vulnerable partly due to gender violence and restricted access to education, employment, and economic independence, conditions that make it difficult to make decisions related to the use of protective methods during sexual intercourse. Several authors have pointed out that machismo and social stereotypes grant men unequal power in sexual relationships, restricting the possibility for women to demand protection. Furthermore, a close association between HIV and intimate partner violence has been documented, which increases the risk of infection, delays diagnosis, hinders access to ART, and reduces therapeutic adherence, which results in a less favorable prognosis in consultations and therapeutic adherence and therefore an unfavorable prognosis ^10^^-^^12^. In this sense, it would be highly relevant to develop new lines of research focused on this population, which differs from those previously studied by other authors.

In relation to oncological diseases, a frequency of cervical cancer in women was observed, while Kaposi’s sarcoma predominated in men, similar to other studies ^13^. These neoplasms, along with non-Hodgkin lymphoma (NHL) ^14^, are classified as AIDS-defining malignancies and are related to oncogenic viruses, which acquire greater virulence and tumor potential in coinfected patients. Likewise, AIDS-defining malignancies have been associated with low CD4 lymphocyte counts ^15^.

Various studies show that, without treatment, HIV causes progressive destruction of the immune system, mainly affecting CD4+ T cells, where the count becomes so low that it increases susceptibility to the development of opportunistic infections and neoplasms. Early initiation of antiretroviral treatment significantly decreases the appearance of these complications ^16^^,^^17^.

On the other hand, psychiatric diseases show a close association with HIV infection, with one of the main risk factors being lack of adherence to treatment, which leads to an increase in viral load and faster disease progression ^18^.

According to the objectives established by UNAIDS, one of the fundamental pillars in HIV care is timely diagnosis and treatment. In the studied population, an area for improvement was identified, given that 25% of patients presented an initial CD4 count of less than 200, and 37.4% did not have this value recorded, which indicates lower-than-expected compliance compared to global epidemiological data ^5^. Among the main limitations of the present study is the exclusive use of retrospective data obtained from clinical records. This methodology prevented the direct collection of information from patients, resulting in missing data for key variables. Specifically, information could not be obtained on sexual orientation in 127 patients, initial CD4 count in 326 patients, and the last CD4 count in 23 patients. Likewise, there was an absence of data on viral load in 22 patients and the reason why 6 patients were not receiving ART at the time of the study.

In relation to the scope of ART, it was observed that 99% of patients with HIV received it, thus exceeding expectations regarding the UNAIDS objectives. Furthermore, the studied population achieved an 88.1% undetectable viral load, which remains an area of opportunity to improve this process. It should be noted that 1.6% who received ART experienced treatment failure; hence the importance of generating strategies that allow for timely diagnosis and treatment.

With the introduction of new antiretroviral therapies, life expectancy has increased over the years ^19^, so much so that currently, patients who initiate ART with high CD4 levels have a life expectancy similar to the general population ^5^. In our population, despite initiating ART in patients with heterosexual preference and other unspecified preferences, the time since disease diagnosis has been more than 11 years, while in those with homosexual and bisexual preference it has been 6 to 11 years, which suggests that life expectancy has increased, making it interesting to conduct research on this in the Mexican population. Despite the high percentage of patients who received ART, the main limitations of the study lie in the lack of information about the reasons why six patients are not currently receiving said treatment, as well as the lack of knowledge about the reasons why 7.6% presented detectable viral load and 7.5% a low CD4 count. These questions remain as future lines of research.

It should be mentioned that the follow-up of these patients occurred during the COVID-19 pandemic, which may have contributed to late diagnoses and faster progression of comorbidities, including oncological ones. A study in Chile by Soto Silva 2022 reinforces this observation by documenting that the epidemiological situation of HIV infection worsened in his country during the pandemic ^20^.

In conclusion, the young, single, homosexual male population is vulnerable to HIV infection. These findings highlight the need to strengthen prevention programs, timely diagnosis, and early treatment to improve clinical outcomes and reduce HIV transmission.
