# Supplementary material for: Epidemiology and treatment in patients with HIV in a secondary-level hospital in Mexico
Source: Rev Peru Med Exp Salud Publica. 2025 Dec 10;42(4):409–15. doi: 10.17843/rpmesp.2025.424.14693 (PMC12879980; doi:10.17843/rpmesp.2025.424.14693)
Supplement: Translation

# Translation: Epidemiología y tratamiento en pacientes con VIH en un hospital de segundo nivel en México

**Authors:** María Fernanda Aguilar-Etchegaray, Hector Martinez-Troncoso, Lilia Susana Gallardo-Vidal, Brenda Michelle Pérez-García, Jacqueline Jiménez-Avila

**Affiliations:** 1 Unidad de Medicina Familiar N°13, Instituto Mexicano del Seguro Social, Querétaro, México.; 2 Hospital General Regional No.1, Instituto Mexicano del Seguro Social, Querétaro, México.

**Keywords:** Epidemiología, Seroprevalencia de VIH, Tratamiento Antirretroviral Altamente Activa

## Abstract

Con el objetivo de describir las características sociodemográficas, clínicas y terapéuticas de los pacientes con Virus de Inmunodeficiencia Humana) (VIH) en un hospital de segundo nivel en México, se realizó un estudio descriptivo basado en la revisión y análisis de 871 expedientes clínicos del 2017 al 2023. Los resultados muestran un predominio del sexo masculino (91,5%) con edades entre 30 y 39 años (45,5%), y orientación sexual homosexual (68,0%). El 25,4% tuvo un recuento CD4 inicial <200 células, el 99% recibió terapia antirretroviral (TAR), posteriormente el 54,5% tuvo un recuento CD4 >500 células, el 88,1% presentó carga viral indetectable y 1,6% presentó falla al tratamiento. En conclusión, la población masculina homosexual joven y soltera es vulnerable para el contagio del VIH. Estos hallazgos resaltan la necesidad de fortalecer los programas de prevención, diagnóstico oportuno y tratamiento precoz, para mejorar los resultados clínicos y reducir la transmisión del VIH.

## INTRODUCCIÓN

A pesar de los cambios epidemiológicos significativos en estos últimos años sobre contagio por el virus de la inmunodeficiencia humana (VIH), en el mundo ^1^ y en México sigue siendo un problema de salud pública ^2^^-^^4^. Ante esta situación, la Organización Mundial de la Salud sobre el VIH-SIDA (ONU-SIDA) instituyó para el año 2020 objetivos precisos de mejora de los pacientes con VIH denominados “objetivos 90/90/90” para garantizar al 90% de las personas un diagnóstico serológico, tratamiento antirretroviral (TAR) y una carga viral indetectable ^5^^-^^6^.

A pesar de los avances logrados con la TAR, la implementación generalizada de tabletas únicas y el manejo inyectable de acción prolongada^3^ se ha visto obstaculizada. Aunque estos fármacos se consideran más eficaces, mejor tolerados y promueven un mayor apego, su introducción aún no permite alcanzar el objetivo final propuesto.

Esta situación se refleja en los datos del año 2022, donde solo: el 86% de las personas conocía su estado serológico; el 76% tenía acceso al TAR; el 71% tenía una carga viral indetectable ^5^.

En México, uno de los mayores retos para enfrentar el VIH es el diagnóstico y tratamiento oportuno, desafortunadamente sólo se cuenta entre el 80 y 85% de cobertura. Por tanto, uno de los mayores desafíos para enfrentar el VIH en México es la detección oportuna, y que todas las personas que lo padecen se encuentren con TAR ^2^.

Según ONU-SIDA, en México, las poblaciones con más incidencia son aquellas más vulnerables como los hombres que tienen relaciones sexuales con hombres (HSH), las personas transgénero (TG), las trabajadoras sexuales, aquellas que usan drogas intravenosas y personas privadas de su libertad ^6^^-^^8^, similar a poblaciones de otros países ^(^^4^^,^^8^, y en la mayoría de los casos no se realiza un diagnóstico ni tratamiento de manera oportuna, por lo que da como consecuencia estadios avanzados de inmunosupresión, peor respuesta al TAR y un incremento en el riesgo de mortalidad ^9^.

Ante estos retos, el Instituto Mexicano del Seguro Social (IMSS) atendió en el 2021 a 82,716 pacientes infectados por VIH, de estos el 97,4% recibieron TAR y presentaron una carga viral menor a 1000 copias ^2^. En 2022, se establecen pautas de actuación para el personal de salud con el fin de homologar la atención de los pacientes con VIH, optimizar los procesos y garantizar la calidad asistencial mediante protocolos de atención Integral ^2^. No obstante, es necesario desarrollar más investigaciones que profundicen en los aspectos epidemiológicos, diagnóstico oportuno y el tratamiento de estos pacientes. En este contexto, el objetivo del presente estudio fue describir las características epidemiológicas y terapéuticas de los pacientes con VIH en un hospital de segundo nivel en México.

**MENSAJE CLAVE**

Motivación para realizar el estudio: De acuerdo a los objetivos de la ONU-SIDA el 90% de las personas que viven con VIH deben conocer su nivel serológico, recibir tratamiento antirretroviral (TAR) y alcanzar una carga viral indetectable, en México pocos estudios actuales evalúan estos indicadores.

Principales hallazgos: La población masculina joven presenta mayor vulnerabilidad al contagio del VIH. Asimismo, las mujeres heterosexuales constituyen un grupo en riesgo en relación con los mecanismos de transmisión del virus en México. El 99% de los participantes recibió TAR y el 88% logro una carga viral indetectable.

Implicancias. Estos hallazgos amplían el conocimiento actual sobre el comportamiento del VIH en población de Querétaro, México, y subrayan la importancia de la prevención y el diagnóstico oportuno.

## EL ESTUDIO

Se realizó un estudio descriptivo, transversal, en expedientes clínicos de pacientes con VIH que recibieron atención especializada en la clínica de prevención y control de VIH y SIDA en el Hospital Regional General No.1 en el Estado de Querétaro, México durante el periodo del 2017-2023. Se incluyeron mayores de 18 años de edad con diagnóstico confirmado de VIH, 5 expedientes fueron eliminados.

Se analizaron las variables edad, estado civil, escolaridad, sexo y orientación sexual, comorbilidades, y antecedentes de enfermedades; recuento de CD4 células/uL (bajo <200; medio 201-499; alto >500 o sin laboratorios), carga viral (indetectable <40 copias/mL; detectado; falla; sin tratamiento; sin laboratorios), ambos basados en el resultado más reciente registrado en el sistema de notas y expedientes, tiempo de diagnóstico de VIH (<2 años; 3-5 años; 6-11 años o >11 años), e inicio del tratamiento (<1 mes del diagnóstico; de 1 a 12 meses).

Los resultados fueron analizados por medio de frecuencias y porcentajes por medio del programa IBM SPSS Statistics version 2019. El protocolo del estudio fue aprobado por el Comité de Ética e Investigación del IMSS del Hospital General Regional N°1 (HGR N°1) con folio: R-2024-2201-116. Los datos personales de los pacientes fueron protegidos mediante el uso de un folio numérico. Los autores declaran que en este artículo no aparecen datos de pacientes identificables.

## HALLAZGOS

De 871 casos de VIH identificados en el HGR N°1 del IMSS Querétaro México del año 2017 al 2023. Al analizar las características por sexo se observó el predominó masculino 797 (91,5%), el estado civil soltero (74,8%), la edad de 30-39 años (45,5%), escolaridad licenciatura (39,8%), ocupación empleada (61,5), orientación sexual homosexual (68,0%), y en el sexo femenino el estado civil casado (28,4%), edad de 40-49 años (37,8%), la escolaridad secundaria (31,1%), ocupación hogar (40,5%), orientación sexual heterosexual (91,9%).

En relación con las comorbilidades preponderaron las enfermedades infecciosas 503 (57,75%), las enfermedades cutáneas 260 (29,85%), y las psiquiátricas 181 (20,78%). Se observó un predominio de enfermedades oncológicas (27,2%), diabetes (22,97%), y endocrinopatía (12,16%) en el sexo femenino con respecto al masculino (Tabla 1).

**Tabla 1:** Características sociodemográficas y comorbilidades encontradas en los pacientes con VIH por sexo.

Características (n = 871)		Total		Hombres		Mujeres	
		n	%	n	%	n	%
Estado civil							
	No especifica	68	7,8	61	7,7	7	9,5
	Soltero (a)	616	70,7	600	75,1	16	21,5
	Casado (a)	75	8,6	54	6,8	21	28,4
	Unión libre	86	9,9	69	8,7	17	23,0
	Divorciado (a)	7	0,8	6	0,8	1	1,4
	Viudo (a)	19	2,2	7	0,9	12	16,2
Grupo etario							
	20-29	109	12,5	105	13,2	4	5,4
	30-39	378	43,3	363	45,5	15	20,3
	40-49	191	21,9	163	20,5	28	37,8
	50-59	123	14,1	107	13,4	16	21,6
	>60	70	8,0	59	7,4	11	14,9
Escolaridad							
	Analfabeta	82	9,4	68	8,5	14	18,9
	Primaria	35	4,0	23	2,9	12	16,2
	Secundaria	118	13,6	95	11.9	23	31,1
	Bachillerato	286	32,8	266	33,4	20	27,0
	Técnico	29	3,3	28	3,5	1	1,1
	Licenciatura	321	36,9	317	39,8	4	5,4
Ocupación							
	Hogar	84	9,6	54	6,8	30	40,5
	Obrero	65	7,5	57	7,2	8	10,8
	Empleado	518	59,5	490	61,5	28	37,8
	Profesionista	156	17,9	154	19,3	2	2,7
	Comerciante	29	3,3	24	3,0	5	6,8
	Estudiante	19	2,2	18	2,3	1	1,4
Orientación sexual							
	Heterosexual	142	16,3	74	9,3	68	91,9
	Homosexual	543	62,3	542	68,0	1	1,4
	Bisexual	59	6,7	59	7,4	0	0,0
	No especifica	127	14,5	122	15,3	5	6,8
Comorbilidades / enfermedades							
	Hipertensión	87	9,9	76	9,54	11	11,8
	Diabetes	63	7,2	46	5,77	17	22,9
	Nefropatía	61	7,0	57	7,15	4	5,4
	Endocrinopatía	56	6,4	47	5,9	9	12,1
	Psiquiátricas	181	20,7	163	20,5	18	24,3
	Oncológicas	79	9,0	59	7,4	20	27,0
	Cutáneas	260	29,8	236	29,6	24	32,4
	Antecedente de tuberculosis	42	4,8	40	5,0	2	2,7
	Enfermedades infecciosas	503	57,7	462	57,9	41	55,4

El tipo de cáncer más frecuente en el sexo femenino fue el cáncer de cérvix (CC) en el 78.9% y en el sexo masculino fue el sarcoma de Kaposi 35,4%.

Predominó el recuento de CD4 alto en el 54% de los pacientes, seguido del recuento medio en el 35,4%, y la carga viral indetectable en el 88,1%. El 92,8% fue manejado a través de TAR combinada de la transcriptasa inversa análogos de los nucleósidos (ITRAN) + Inhibidor de la transcriptasa inversa no análogos de los nucleósidos (ITRNN) + Inhibidor de la Integrasa (INI) en tableta única, seguido por la combinación ITRAN+ITRNN en 1,6% de los pacientes (Tabla 2).

**Tabla 2:** Recuento de CD4, carga viral y tratamiento antirretroviral recibido por pacientes con VIH por sexo

		Total		Hombres		Mujeres	
		n	%	n	%	n	%
Recuento CD4 inicial							
	Bajo (<200)	221	25,4	204	25.6	17	23,0
	Medio (201-499	219	25,1	199	25,0	20	27,0
	Alto (>500)	105	12,1	99	12,4	6	8,1
	Sin laboratorios	326	37,4	295	37,0	31	41,9
Último recuento CD4							
	Bajo (<200)	65	7,5	59	7,4	6	8,1
	Medio (201-499)	308	35,4	281	35,2	27	36,5
	Alto (>500)	475	54,5	438	55,0	37	50,0
	Sin laboratorios	23	2,6	19	2,4	4	5,4
Carga viral							
	Detectado	66	7,6	60	7,5	6	8,1
	Indetectable	767	88,1	703	88,2	64	86,5
	Falla	14	1,6	13	1,6	1	1,4
	Sin tratamiento	2	0,2	2	0,3	0	0,0
	Sin laboratorio	22	2,5	19	2,4	3	4,1
Única tableta							
	BET/ABC (2 ITRAN+INI)	808	92,8	742	93,1	66	89,2
Múltiples tabletas							
	ITRAN+ITRNN+INI	2	0,2	2	0,3	0	0,0
	RITO+INI+MVC	2	0,2	2	0,3	0	0,0
	ITRAN+IP	5	0,6	4	0,5	1	1,4
	ITRNN+IP+INI	1	0,1	0	0,0	1	1,4
	RITO+INI	1	0,1	1	0,1	0	0,0
	IP+RITO+INI+MVC	2	0,2	2	0,3	0	0,0
	ITRAN+IP+RITO	10	1,1	10	1,3	0	0,0
	ITRAN+ITRNN	14	1,6	13	1,6	1	1,4
	2 ITRAN + IP	2	0,2	2	0,3	0	0,0
	2 ITRAN+IP+RITO+INI	1	0,1	1	0,1	0	0,0
	ITRAN+ITRNN+IP	8	0,9	8	1	0	0,0
	ITRAN+IP+INI	4	0,5	2	0,3	2	2,7
	2 ITRAN+ITRNN	5	0,6	4	0,5	1	1,4
No toma medicamento		6	0,7	4	0,5	2	2,7

ITRAN: Inhibidor de la transcriptasa inversa análogos de los nucleósidos. ITRNN: Inhibidor de la transcriptasa inversa no análogos de los nucleósidos. INI: Inhibidor de la Integrasa. IP: Inhibidor de la proteasa. RITO: Ritonavir. MVC: Maraviroc.

Se hizo un análisis por preferencia sexual y el tiempo de evolución de VIH y se observó que los pacientes con preferencia homosexual 235 (43,3%), y 26 (44,1%) de preferencia bisexual, tuvieron una evolución de 6 a 11 años. En el caso de aquellos con preferencia heterosexual, 61 (43%) tuvieron una evolución de mas de 11 años (Figura 1). Así mismo se analizó la relación del inicio del TAR con la preferencia sexual, y se observó que en el caso de la preferencia homosexual 232 (42,7%), y 54 (38%) pacientes con preferencia heterosexual iniciaron el tratamiento antes del mes de diagnóstico (Figura 2).

**Figura 1:** Tiempo de evolución en pacientes diagnosticados con VIH por preferencia sexual.

**Figura 2:** Inicio de tratamiento antirretroviral en pacientes con VIH por preferencia sexual.

## DISCUSIÓN

Los hallazgos del presente estudio muestran que el sexo masculino con orientación homosexual continúa siendo el grupo más frecuente entre los pacientes con VIH. Al analizar los datos por sexo, se observa que, en las mujeres, predominan las casadas, amas de casa, con mayor edad que los hombres, pero con un nivel educativo inferior.

Actualmente, existe poca literatura sobre los factores asociados a la transmisión del VIH en mujeres heterosexuales, lo que limita la compresión de las características de esta población ^10^^-^^12^. Este grupo se considera vulnerable en parte debido a la violencia de género y el acceso restringido a la educación, al empleo y a la independencia económica, condiciones que dificultan la toma de decisiones relacionadas con el uso de métodos de protección durante las relaciones sexuales. Diversos autores han señalado que el machismo y los estereotipos sociales otorgan a los hombres un poder desigual en las relaciones sexuales, restringiendo la posibilidad de las mujeres de exigir protección. Además, se ha documentado una estrecha asociación entre el VIH y la violencia de pareja, lo cual incrementa el riesgo infección, retrasa el diagnostico, dificulta el acceso al TAR y reduce la adherencia terapéutica, lo que se traduce en un pronóstico menos favorable consultas y a la adherencia terapéutica y por lo tanto un pronóstico desfavorable ^10^^-^^12^. En ese sentido, sería de gran relevancia desarrollar nuevas líneas de investigación enfocadas en esta población, que difiere de las previamente estudiadas por otros autores.

En relación a las enfermedades oncológicas, se observó una frecuencia de cáncer cervicouterino en mujeres, mientras que en los hombres predominó el sarcoma de Kaposi, similar a otros estudios ^13^. Estas neoplasias junto con el linfoma no Hodgkin (LNH) ^14^, se clasifican como neoplasias definitorias de SIDA, y estar relacionados con virus oncogénicos, que adquieren mayor virulencia y potencial tumoral en pacientes coinfectados. Asimismo, las neoplasias definitorias de SIDA se han asociado con recuentos bajos de linfocitos CD4 ^15^.

Diversos estudios muestran que, sin tr atamiento, el VIH provoca una destrucción progresiva del sistema inmunológico, afectando principalmente las células T CD4+, donde el recuento llega a ser tan mínimo, que aumenta la susceptibilidad al desarrollo de infecciones oportunistas y neoplasias. Un inicio temprano del tratamiento antirretroviral disminuye significativamente la aparición estas complicaciones ^16^^,^^17^.

Por otro lado, las enfermedades psiquiátricas muestran una estrecha asociación con la infección por VIH, siendo uno de los principales factores de riesgo la falta de adherencia al tratamiento, que conlleva un incremento de la carga viral y una progresión más rápida de la enfermedad ^18^.

De acuerdo a los objetivos establecidos por la ONUSIDA, uno de los pilares fundamentales en la atención del VIH es el diagnostico tratamiento oportuno. En la población estudiada se identificó un área de mejora, dado que el 25% de los pacientes presentaron un recuento inicial de CD4 menor a 200, y el 37,4% no se registró dicho valor, lo que indica un cumplimiento inferior al esperado en comparación con los datos epidemiológicos globales ^5^. Entre las limitaciones principales del presente estudio se encuentra el uso exclusivo de datos retrospectivos obtenidos de expedientes clínicos. Esta metodología impidió la recolección directa de información de los pacientes, resultando en datos faltantes para variables clave. Específicamente, no se pudo obtener información sobre la orientación sexual en 127 pacientes, el recuento inicial de CD4 en 326 pacientes, y el último recuento de CD4 en 23 pacientes. Asimismo, hubo ausencia de datos sobre la carga viral en 22 pacientes y la razón por la cual 6 pacientes no recibían TAR en el momento del estudio.

En relación al alcance del TAR, se pudo observar que el 99% de los pacientes con VIH, lo recibieron, por lo que se supera la expectativa con respecto a los objetivos de la ONUSIDA. Además la población estudiada obtuvo un 88,1% de carga viral indetectable, lo que sigue siendo un área de oportunidad para mejorar este proceso. Cabe referir que el 1,6% que recibió TAR presentó falla al tratamiento; de ahí la importancia de generar estrategias que permitan realizar un diagnóstico y tratamiento oportuno.

Con la introducción de nuevas terapias antirretrovirales, la esperanza de vida se ha incrementado con el paso de los años ^19^, tanto que en la actualidad aquellos pacientes que inician TAR con niveles elevados de CD4 la esperanza de vida es similar a la población en general ^5^. En nuestra población a pesar de iniciar TAR en pacientes con preferencia heterosexual y otras preferencias no especificadas el tiempo de evolución de la enfermedad ha sido a más de 11 años, mientras que, en aquellos con preferencia homosexual y bisexual de 6 a 11 años, lo que hace pensar que la esperanza de vida se ha incrementado, por lo que sería interesante poder llevar una investigación al respecto en población mexicana. A pesar del alto porcentaje de pacientes que recibieron TAR, las limitaciones principales del estudio radican en la falta de información sobre las causas por las cuales seis pacientes no reciben actualmente dicho tratamiento, así como el desconocimiento de los motivos por los que el 7,6% presentó carga viral detectable y el 7,5% un recuento de CD4 bajo. Estas cuestiones quedan como líneas de investigación futuras.

Cabe referir que el seguimiento de estos pacientes se dio durante el tiempo de pandemia por COVID-19, que pudo haber contribuido a diagnósticos tardíos y progresión más rápida de comorbilidades, incluidas las oncológicas. Un estudio en Chile por Soto Silva 2022 refuerza esta observación al documentar que la situación epidemiológica de la infección por VIH empeoró en su país durante la pandemia ^20^.

En conclusión la población masculina homosexual joven y soltera es vulnerable para el contagio del VIH. Estos hallazgos resaltan la necesidad de fortalecer los programas de prevención, diagnóstico oportuno y tratamiento precoz, para mejorar los resultados clínicos y reducir la transmisión del VIH.
